# Severe malaria in Battambang Referral Hospital, an area of multidrug resistance in Western-Cambodia: a retrospective analysis of cases from 2006–2009

**DOI:** 10.1186/1475-2875-12-217

**Published:** 2013-06-27

**Authors:** Chanthap Lon, Ans Timmermans, Nillawan Buathong, Samon Nou, Youry Se, Ngo Sitthy, Soklyda Chann, Somporn Kraesub, Tippa Wongstitwilairoong, Douglas S Walsh, Stuart Tyner, Mark Fukuda, David Callender, Jeffrey Sherwood, Lenin Koy, Mengchour Char, Delia Bethell, David Saunders

**Affiliations:** 1 Department of Immunology & Medicine, Armed Forces Research Institute of Medical Sciences, 315/6 Rajvithi Road, Bangkok 10400, Thailand; 2 Armed Forces Research Institute of Medical Sciences, Phnom Penh, Cambodia; 3 Battambang Referral Hospital, Cambodia, Dongkorteap village, Tuol Ta Ek Commune, Battambang District, Battambang Province, Cambodia; 4 Armed Forces Health Surveillance Center, Silver Spring, MD, USA; 5 Tripler Army Medical Center, Honolulu, HI, USA; 6 William Beaumont Army Medical Center, El Paso, TX, USA; 7 National Center for Parasitology, Entomology and Malaria Control, #372 Blvd. Monivong, Phnom Penh, Cambodia

## Abstract

**Background:**

Despite recent malaria containment and control efforts leading to reduced incidence, Cambodia remains endemic for both *Plasmodium vivax* and multidrug-resistant *Plasmodium falciparum* malaria. Little has been reported in the peer-reviewed literature regarding the burden of severe malaria (SM) in Cambodia.

**Methods:**

Medical records for all patients admitted to the Battambang Referral Hospital (BRH) with an admitting or discharge diagnosis of SM from 2006 to 2009 (suspected SM cases) were reviewed. Those meeting the case definition of SM according to retrospective chart review and investigator assessment of probable cases, based on published national guidelines available at the time, were analysed for trends in demographics, mortality and referral patterns.

**Results:**

Of the 537 suspected SM cases at BRH during the study period, 393 (73%) met published WHO criteria for SM infection. Despite limited diagnostic and treatment facilities, overall mortality was 14%, with 7% mortality in children 14 and under, but 19% in adults (60% of cases). Cerebral malaria with coma was relatively rare (17%), but mortality was disproportionately high at 35%. Mean time to hospital presentation was five days (range one to 30 days) after onset of symptoms. While patients with delays in presentation had worse outcomes, there was no excess mortality based on treatment referral times, distance travelled or residence in artemisinin-resistance containment (ARC) Zone 1 compared to Zone 2.

**Conclusions:**

Despite limitations in diagnosis and treatment, and multiple confounding co-morbidities, mortality rates at BRH were similar to reports from other countries in the region. Interventions to improve access to early diagnosis and effective treatment, combined with modest improvements in intensive care, are likely to reduce mortality further. Patients referred from Zone 1 did not have excess mortality compared to Zone 2 ARC areas. A steep decrease in SM cases and deaths observed in the first half of 2009 has since continued, indicating some success from containment efforts despite the emergence of artemisinin resistance in this area.

## Background

By definition, patients with severe malaria are in critical condition and require emergency therapy with parenteral drugs [1]. Untreated, mortality approaches 100%. Little has been reported in the peer-reviewed literature about the burden of severe malaria in Western Cambodia, located in World Health Organization’s (WHO) Zone 1 artemisinin resistance containment (ARC) area.

Through implementation of a successful national malaria control programme, officially reported malaria incidence in Cambodia decreased by more than 50% between 2001–2010, from 11.03 cases per 1,000 in 2000 to 4.07 cases per 1,000 in 2010. Malaria mortality steadily decreased over the last decade from 5.2 deaths (in 2001) to 0.98 deaths per 100,000 in 2010 [2]. Despite these successes, 60% of Cambodia's territory continues to be endemic for malaria, though much of this is sparsely populated. The majority of cases occur in eastern provinces, with artemisinin resistance emerging in the west.

**Figure 1:**
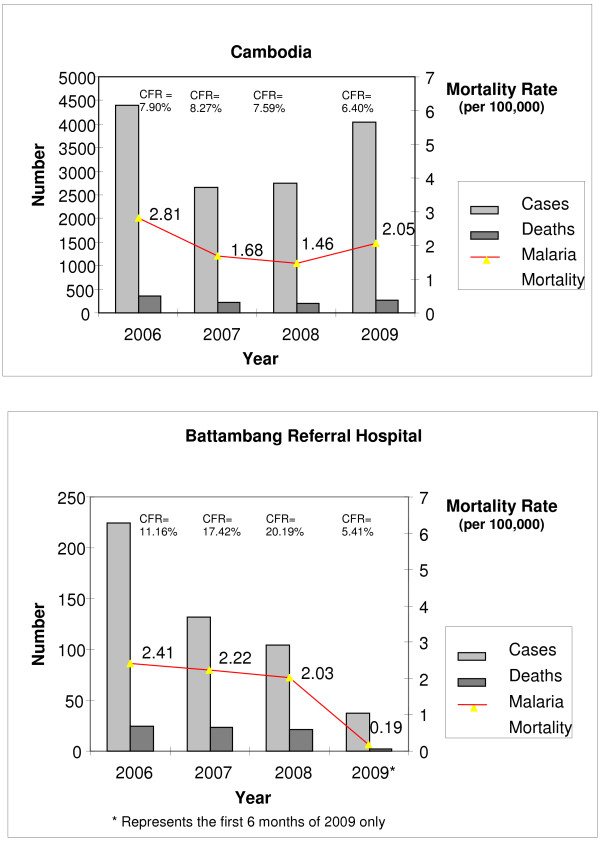
Morbidity, mortality (number of deaths per 100,000 per year) and case fatality rates (CFR, % deaths) from severe malaria in referral hospitals between 2006–9, Cambodia versus Battambang Referral Hospital.

In 2007 an estimated 2.13 million Cambodians (about 15% of the total population) living within 2 km of an endemic forested area were at risk of malaria, with 42,124 confirmed cases. There were 2,648 severe cases admitted to referral hospitals, of which 219 died. Data from the National Center for Parasitology, Entomology and Malaria Control, Cambodia (CNM) in 2007 indicated that Battambang Province had the second highest case-fatality rate (CFR) nationally (17.4%) – double the national CFR (Figure 1).

Study objectives were to assess incidence of severe malaria at a tertiary referral hospital, and further characterize the presentation of severe malaria in this resource-limited setting based on WHO recommended case definitions. A priori, it was suspected that access to pre-hospital care, drug availability and compliance, and delayed presentation would be important factors in outcomes and survival. Differences in mortality from the two separate ARC zones served by Battambang Referral Hospital (BRH) were assessed. The operational utility of published guidelines for the diagnosis and management of severe malaria were also evaluated. The authors report here the first data on the burden of severe malaria at the government referral hospital in Battambang, Cambodia’s second largest city, and the first area in the world to report artemisinin-resistant malaria [3].

## Methods

### Study location

**Figure 2:**
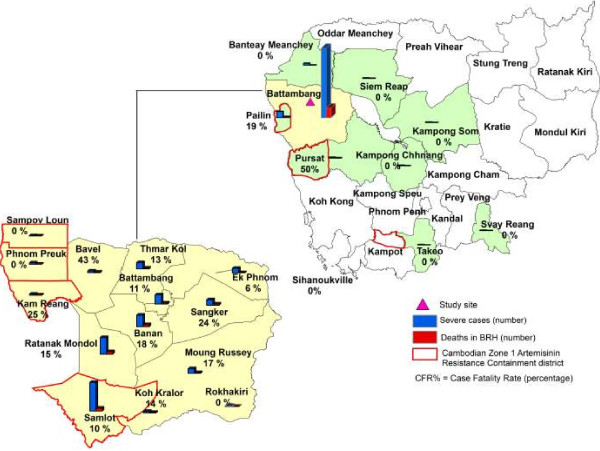
Map of Cambodia with study site location and case fatality rates according to province of residence/origin, with inset showing a map of Battambang Province with case fatality rates according to district of residence.

The study was conducted at the BRH, located in Battambang City in Battambang Province, Western Cambodia (Figure 2). BRH is a 270-bed facility and the main hospital for Battambang city and surrounding districts and admits on average 2,000 patients per month.

### Identification of cases

Medical records for all patients admitted to the BRH from January 2006 to June 2009 with an admission and/or discharge diagnosis of severe malaria (irrespective of confirmation by slide or rapid diagnostic test) were reviewed to analyse morbidity, mortality, demography and referral patterns. Since no separate log for severe malaria cases existed, all hospital records for the study period were searched in order to identify the severe malaria cases. Individual patient records were reviewed by a study physician, and data systematically extracted onto case report forms. Discharge diagnoses were reviewed independently for adherence to the Cambodian national diagnosis and treatment guidelines in use at the time. Severe malaria was defined as a complicated case of malaria infection (*Plasmodium falciparum* and/or *Plasmodium vivax*) presenting with one or more criteria of severe disease [1]. WHO criteria for severe malaria were adopted by the Ministry of Health as part of the National Malaria Treatment Guidelines in Cambodia and all clinicians would have had access to these guidelines at the time of the study. The availability of means to assess the various diagnostic and prognostic parameters is outlined in Table 1.

**Table 1:** Availability of means to assess diagnostic and prognostic parameters, and provide treatment of severe malaria at Battambang Referral Hospital

**WHO severe malaria criteria**	**Description as published in national guidelines**	**Availability at BRH**	**Treatment available**
**Prostration**	**Inability to sit upright without support or to drink**	**Yes**	**Supportive only**
Impaired consciousness	Modified Glasgow score ≤ 9 in adult and children >5 years old or Blantyre coma score ≤ 2/5 in children who have not learned to speak	Yes	Supportive only
**Respiratory distress**	**Sustained nasal flaring, intercostals recession and deep or fast breathing (RR >25/min in adults and >40/min in children)**	**Yes**	**Limited - no ventilators, low flow oxygen only**
Multiple convulsions	> 1/24 h	Yes	Parenteral or NG diazepam
**Circulatory collapse**	**BP <50 mmHg before 5 years old or BP <80 mmHg above 5 years old, cool peripheries, weak pulses**	**Yes**	**Peripherally administered IV pressors, fluids**
Pulmonary oedema	Radiological features	Yes	Limited - diuretics, oxygen, morphine
**Abnormal bleeding**	**(No definition provided)**	**Yes**	**Blood transfusion (limited)**
Jaundice	(No definition provided)	Yes	Supportive
**Haemoglobinuria**	**(No definition provided)**	**Yes**	**Blood transfusion (limited)**
Frequent vomiting	Vomiting everything with inability to retain food or medicines	Yes	Antiemetic drugs, parenteral route of drug administration
**Laboratory finding**			
Severe anaemia (or pallor)	Hb <5g/dl or Ht <15%	Yes	Blood transfusion (limited)
**Hypoglycaemia**	**Glycaemia <2.2 mmol/l or 0.4 g/l**	**Yes**	**IV dextrose**
Metabolic acidosis	Plasma bicarbonate <15 mmol/l	No	IV fluids
**Hyperlactataemia**	**Plasma lactate >5 mmol/l**	**No**	**IV fluids**
Hyperparasitaemia	*P*. *falciparum* “++++” or >200 000 parasites/**μ**l	Often	Intramuscular artemether
**Renal failure (or oliguria)**	Urine output <400 ml/24 h or plasma creatinine>265 **μ**mol/l in adult or urine output <12 ml/kg/24 h or plasma creatinine above the age-related normal range, persisting after rehydration in children	Clinical only - rare lab tests	Peritoneal dialysis occasionally available; supportive care

For the purpose of this paper, referral to ‘smear-positive’ patients indicates those with a presence of asexual malaria parasites as detected by microscopy or rapid diagnostic test. Overall CFR and number of cases who died included those dying in hospital, as well as those who were discharged to home in worsening (moribund) condition. The retrospective hospital record review was carried out in compliance with the Declaration of Helsinki 2000, following protocol approval by the Walter Reed Army Institute of Research IRB and the National Ethical Committee for Health Research (NECHR) in Cambodia. Personal identifying information was not collected on the case report forms.

### Data analysis

Data were translated where needed from French and/or Khmer into English, and transcribed from medical records onto paper case report forms, and analysed using SPSS 12.0.0 for Windows. Double data entry was performed using Microsoft Excel to minimize transcription errors. Descriptive statistical analyses were performed for demography, referral patterns, symptoms, signs, admitting laboratory parameters, complications with respect to survival outcomes, and case management data. Fisher’s exact test (two-sided) was used to compare proportions from non-parametric tests when the expected values were less than five. Mortality risk was expressed with odds ratios (OR) and 95% confidence intervals (95% CI). Multiple logistic regression analysis was used to calculate adjusted odds ratios (aOR) to evaluate associations of mortality with presenting signs and symptoms, and complications occurring during hospital admission. Variables with crude OR significant at the level of p <0.2 in univariate analyses were included in a multivariate analysis.

## Results

**Figure 3:**
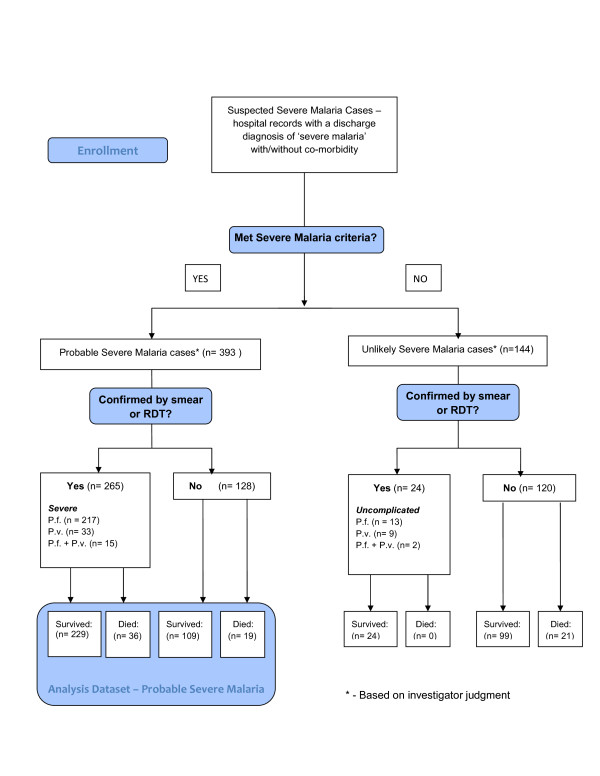
Study flow diagram.

Between January 2006 and June 2009, a total of 537 records from suspected severe malaria cases were reviewed from patients admitted or discharged from BRH with a diagnosis of severe malaria. After review by study physicians, 393 patients met published Cambodian severe malaria case definitions and were considered to be ‘probable severe malaria cases’ (Figure 3).

### Demographic and clinical characteristics and mortality

Overall CFR among the 393 probable severe malaria cases was 14.0% (55 out of 393), with the majority dying (38 or 9.7%) in hospital. The majority were male (224 or 57.0%), aged between five and 30 (median age 18 years), and were school-aged children (107 or 27%) or farmers (101 or 26%). Most patients (92%) were self-referred, arrived by private vehicle, and resided in Battambang Province (Table 2). Among the 393 probable severe malaria cases, 265 (67%) had malaria diagnoses confirmed by blood smear or rapid diagnostic test (RDT). Malaria species identified in the 265 smear/RDT confirmed cases included 217 *P*. *falciparum* (81.9%), 33 *P*. *vivax* (12.4%); and 15 documented mixed infections (5.7%) positive for both *P*. *falciparum* and *P*. *vivax* (Table 2).

**Table 2:** Demographic and clinical characteristics of patients that met severe malaria criteria (n=393)

	**Slide positive**					**Slide negative/Not done**				
	**Total**	**Survived**		**Died**		**Total**	**Survived**		**Died**	
	**n**	**n**	**(%)**	**n**	**(%)**	**n**	**n**	**(%)**	**n**	**(%)**
**Age category**										
0-4	30	28	(93)	2	(7)	23	18	(78)	5	(22)
5-14	72	70	(97)	2	(3)	41	36	(88)	5	(12)
15-30	89	70	(79)	19	(21)	27	24	(89)	3	(11)
31-49	53	44	(83)	9	(17)	26	22	(85)	4	(15)
>=50	21	17	(81)	4	(19)	11	9	(82)	2	(18)
**Sex**										
Male	152	133	(88)	19	(13)	72	62	(86)	10	(14)
Female	113	96	(85)	17	(15)	56	47	(84)	9	(16)
**Occupation**										
Labour	40	30	(75)	10	(25)	7	6	(86)	1	(14)
Government	3	2	(67)	1	(33)	2	2	(100)	0	(0)
Housewife	11	8	(73)	3	(27)	6	4	(67)	2	(33)
Farmer	66	52	(79)	14	(21)	35	30	(86)	5	(14)
Business	2	2	(100)	0	(0)	1	1	(100)	0	(0)
Monk	1	1	(100)	0	(0)	1	1	(100)	0	(0)
School-aged child	74	69	(93)	5	(7)	33	29	(88)	4	(12)
Unemployed	15	14	(93)	1	(7)	6	5	(83)	1	(17)
Other	48	46	(96)	2	(4)	36	30	(83)	6	(17)
**Referral by**										
Self	173	152	(88)	21	(12)	79	72	(91)	7	(9)
Private sector	1	1	(100)	0	(0)	11	7	(64)	4	(36)
Government Referral Hospital	9	6	(67)	3	(33)	37	29	(78)	8	(22)
Government Health Centre	82	70	(85)	12	(15)	1	1	(100)	0	(0)
**Arrived by**										
Private vehicle	181	157	(87)	24	(13)	76	69	(91)	7	(9)
Government ambulance	78	67	(86)	11	(14)	46	35	(76)	11	(24)
**Parasite species**										
Pf	217	185	(85)	32	(15)					
Pv	33	29	(88)	4	(12)					
Mixed Pf+Pv	15	15	(100)	0	(0)					
Negative						110	94	(85)	16	(15)
ND						18	15	(83)	3	(17)
**Parasitaemia time 0**										
No or low (1+ to 3+)	145	122	(84)	23	(16)	110	94	(85)	16	(15)
Hyperparasitaemia	113	101	(89)	12	(11)	0	0	(0)	0	(0)
(4+ to 6+)										

Out of 55 total deaths reported, there were 36 deaths among the 265 smear-positives (mortality rate 13.6%), versus 19 deaths among the 128 smear-negatives (14.9% mortality). Lowest case fatalities were found in the five to 14 year age group (6.2%), while most fatalities were 15 to 30 years old (19%) or older than 50 (19%). The under-five mortality rate was 13%. Occupations with the highest CFR included labourers (23%), government workers (20%), farmers (19%), and housewives (29%). A higher death rate was found in referred patients (19.2%), in particular those referred from government health facilities, versus patients who came directly to the hospital (8%). There were two fatalities among the 13 pregnant severe malaria cases (both *P*. *falciparum* infections), as well as one spontaneous abortion and three stillbirths (all *P*. *falciparum*). The only pregnant *P*. *vivax* case survived with normal delivery at term during hospital admission.

### Signs and symptoms on admission

The most common presenting signs and symptoms among 393 probable severe malaria cases included fever (95%), headache (63%), dark urine (56%), chills (53%), and impaired consciousness (42%). There were 65 patients who presented in a coma (17%). The only presenting symptoms, adjusted for age group (aOR age >15 years=2.0, 95% CI 0.9-4.1) that remained significantly associated with a fatal outcome after a multiple logistic regression analysis were coma (aOR=5.8, 95% CI 2.9-11.5), pallor (aOR=4.2, 95% CI 1.9-9.0), dyspnea (aOR= 2.5, 95% CI1.2, 5.4) and dark urine (aOR=0.4, 95% CI 0.2-0.8) (Table 3).

**Table 3:** Signs and symptoms on admission in probable severe malaria patients (n= 393)

**Sign/symptoms**	**Total**		**Died**		**OR (95% CI)**	p-value*
	**n**	**(%)**	**n**	**(%)**		
Fever	372	(95)	50	(13)	0.5 (0.2, 1.4)	0.19
Headache	247	(63)	32	(13)	0.8 (0.4, 1.4)	0.46
Dark urine	220	(56)	24	(11)	0.6 (0.3, 1.0)	**0.06**
Chills	207	(53)	26	(13)	0.8 (0.4, 1.4)	0.47
Impaired	166	(42)	23	(14)	1.0 (0.6, 1.8)	1
consciousness						
(excl coma)						
Abdominal pain	133	(34)	15	(11)	0.7 (0.4, 1.3)	0.29
Vomiting	104	(26)	10	(10)	0.6 (0.3, 1.2)	0.14
Anorexia	77	(20)	6	(8)	0.5 (0.2, 1.1)	0.1
Fatigue	75	(19)	8	(11)	0.7 (0.3, 1.5)	0.46
Coma	65	(17)	23	(35)	5.0 (2.7, 9.4)	**0**
Dyspnea	59	(15)	14	(24)	2.2 (1.1, 4.4)	**0.03**
Pallor	50	(13)	19	(38)	5.2 (2.7, 10.2)	**0**
GI Bleeding	41	(10)	4	(10)	0.6 (0.2, 1.9)	0.63
Dizziness	35	(9)	4	(11)	0.8 (0.3, 2.3)	0.8
Bleeding	26	(7)	5	(19)	1.5 (0.5, 4.2)	0.39
Nausea	25	(6)	4	(16)	1.2 (0.4, 3.6)	0.77
Jaundice	24	(6)	4	(17)	1.2 (0.4, 3.8)	0.76
Sweating	24	(6)	6	(25)	2.2 (0.8, 5.8)	0.13
Convulsion	20	(5)	5	(25)	2.2 (0.8, 6.2)	0.18
Confusion	11	(3)	0	(0)	-	-
Neck stiffness	2	(1)	1	(50)	6.2 (0.4, 101)	0.26

*Fisher's Exact Test (2-sided).

Adjusted for Dark Urine, Coma, Dyspnea and Pallor and Age Group, aOR (95% CI) of Dark Urine= 0.4 (0.2, 0.8), aOR of Coma = 5.8 (2.9, 11.5), aOR of Dyspnea = 2.5 (1.2, 5.4), aOR of Pallor = 4.2 (1.9, 9.0) and aOR of Age > 15 = 2.0 (0.9, 4.1).

### Laboratory data

Laboratory assessment at BRH was limited and included microscopy for parasitaemia (94%), white blood cell count (92%), haematocrit (63%) or haemoglobin (6%), platelets (48%) and plasma glucose (32%). Assessment of liver enzymes (3%) were done infrequently, and other commonly used tests including blood urea nitrogen, creatinine, bilirubin, albumin, plasma lactate and bicarbonate were unavailable. Higher parasitaemia based on the qualitative scoring system used was not associated with higher mortality. Although severe anaemia was common, with approximately 29% of patients having haematocrit values less than 15% at initial presentation, mean presenting haematocrit values between non-survivors and survivors were similar (25.2% versus 24.0%), and no patient had a presenting glucose level less than 40 mg/dL. Non-survivors had higher mean leukocyte counts than survivors (10.6 versus 8.0x 103 cells/ml, p-value =0.02). Of 191 patients tested, the majority had a platelet count greater than 150,000/μL, with the mean platelet count significantly lower in non-survivors (183,000/μL versus 208,000/μL, p-value = 0.04) (Table 4).

**Table 4:** Laboratory values reported on admission in probable severe malaria patients (n= 393)

**Laboratory test**	**Total evaluated**			**Mean (S.D)**			**Reference range**	
	**n**	**(%)**	**Range**	**Died**	**Survived**	p-value*	**Category**	**n**
WBC (× 10^3^cells/ml)	368	(94)	1.6-35.2	**10.6 ± 7.3**	**8.0 ± 4.8**	**0.02**	≤ 12	303
							> 12	65
Hematocrit (%)	253	(64)	5.0-50	25.2 ± 10.4	24.0 ± 10.4	0.5	≤ 15	74
							16 to 30	109
							≥ 31	70
Hemoglobin (g/dl)	22	(6)	3.0-16.2	9.8 ± 2.6	9.1 ± 4.2	0.8	< 10	12
							≥ 10	10
Platelets (× 10^3^cells/ml)	191	(49)	10-450	**183 ± 49.4**	**208 ± 47.4**	**0.04**	≤ 100	2
							101-149	11

							≥ 150	178
Glucose (mg/dl)	129	(33)	50-459	102 ± 45.9	113 ± 73.4	0.4	41-60	10
							61-80	51
							81-126	38
							> 126	30
SGOT (IU/L)	11	(3)	19-390	144 ± 154.5	123 ± 126	0.8	< 3 times ULN	6
							≥ 3 times ULN	5
SGPT (IU/L)	11	(3)	20-430	66±45	110 ± 137	0.6	< 3 times ULN	7
							≥ 3 times ULN	4
		**Total evaluated**						**Reference range**
	**n**	**(%)**	**Range**	**# (%) Died**	**# (%) Survived**	p-value**	**Category**	**n**
Parasitaemia	368	(94)	0 to +6	16 (15)	94 (85)	0.4	Negative	110
				23 (16)	122 (84)		1-3+	145
				12 (11)	101 (89)		≥ 4+	113

p< 0.05, *t-test (2-sided) or **One-Way ANOVA.

### Clinical course and complications

Features of the clinical course, and complications that developed during hospital admission are shown in Table 5 (all cases) and Table 6 (children <15 years old), along with their association with mortality. In patients who met the severe malaria case definition (all age groups), the most common clinical features included prostration (68%), impaired consciousness (65%), and respiratory distress (48%), and all three were associated with a substantially increased risk of mortality in a univariate analysis. Circulatory collapse (15%), renal failure (27%), and pulmonary oedema (2%), though less common, were also associated with increased mortality, while severe anaemia defined as a haematocrit of less than 15 (55%) and hyperparasitaemia (33%) were not. In multivariate analysis, only circulatory collapse (aOR67.5; 95% CI 23.2-196) and renal failure (aOR19.7; 95% CI 4.6-83.9) remained statistically significantly associated with a higher mortality risk independent of age. Clinical features were similar in the under 15 age group, although the majority (60%) developed multiple convulsions during their hospital stay. Circulatory collapse (adjusted OR= 186, 95% CI 30.4, 1,143) was the only complication significantly associated with mortality in multivariate logistic regression analysis in the younger age group.

**Table 5:** Clinical course and complications experienced during hospital admission in probable severe malaria patients (n=393)

	**Neg**	**Pf**	**Pv**	**Pf+Pv**	**Not recorded**	**Total**		**Died**		**OR (95% CI)**	p-value*
			**n**			**n**	**(%)**	**n**	**(%)**		
Prostration	82	150	16	5	12	265	(68)	53	(20)	15.6 (3.7, 65.2)	0
Impaired consciousness	81	143	14	5	11	254	(65)	53	(21)	18.1 (4.3, 75.4)	0
Respiratory distress	55	105	12	5	9	186	(48)	39	(21)	3.9 (2.0, 7.5)	0
Severe anaemia	55	90	11	3	10	169	(55)	27	(16)	1.1 (0.6, 2.1)	0.75
Hyperparasitaemia	1	101	7	11	8	128	(33)	13	(10)	0.6 (0.3, 1.1)	0.12
Jaundice	21	38	4	1	4	68	(17)	14	(21)	1.7 (0.9, 3.5)	0.12
Abdominal bleeding	23	29	5	0	5	62	(16)	12	(19)	1.6 (0.8, 3.2)	0.23
Frequent vomiting	17	34	6	3	1	61	(16)	5	(8)	0.5 (0.2, 1.3)	0.23
Circulatory collapse	17	32	7	1	3	60	(15)	43	(72)	67.5 (30.2, 150.8)	0**
Renal failure	8	16	2	0	1	27	(7)	16	(59)	16.8 (7.1, 39.7)	0**
Multiple convulsion	7	11	2	2	2	24	(6)	7	(29)	2.8 (1.1, 7.1)	0.03
Macroscopic	6	6	0	0	0	12	(3)	3	(25)	2.5 (0.7, 9.7)	0.17
haemoglobinuria											
Hypoglycemia	1	7	0	2	0	10	(7)	3	(30)	1.5 (3.6, 6.2)	0.7
Pulmonary oedema	2	2	1	0	0	5	(2)	4	(80)	42.3 (4.5, 398)	0

*Fisher's Exact Test (2-sided).

**Adjusted for age group, circulatory collapse and renal failure, aOR (95% CI) of circulatory collapse=67.5 (23.2, 196) and aOR of renal failure=19.7 (4.6, 83.9).

**Table 6:** Clinical course and complications experienced during hospital admission in probable severe malaria patients for only those aged 0 to 15 years (n=130)

	**Neg**	**Pf**	**Pv**	**Pf+Pv**	**Not recorded**	**Total**		**Died**		**OR (95% CI)**	p-value*
			**n**			**n**	**(%)**	**n**	**(%)**		
Prostration	51	65	8	2	4	130	(78)	13	(10)	3.8 (0.5, 29.9)	0.31
Impaired consciousness	51	65	8	2	4	130	(78)	13	(10)	3.9 (0.5, 30.8)	0.31
Multiple convulsion	3	83	12	1	1	100	(60)	1	(1)	2.9 (0.3, 27.4)	0.36
Severe anaemia	36	51	5	2	3	97	(58)	10	(10)	1.4 (0.4, 4.8)	0.77
Respiratory distress	26	34	4	3	3	70	(42)	11	(16)	8.7 (1.9, 40.5)	0
Hyperparasitaemia	1	33	1	5	1	41	(25)	1	(2)	0.2 (0.03, 1.6)	0.12
Frequent vomiting	14	17	3	2	1	37	(22)	1	(3)	0.2 (0.03, 2.0)	0.2
Abdominal bleeding	12	9	0	0	0	21	(13)	3	(14)	2.0 (0.5, 7.7)	0.4
Circulatory collapse	8	3	1	7	0	19	(11)	10	(53)	186 (30.4, 1143)	0**
Jaundice	6	4	0	0	0	10	(6)	2	(20)	3.0 (0.6, 15.8)	0.2
Macroscopic haemoglobinuria	3	0	0	0	0	3	(2)	1	(33)	5.7 (0.5, 66.6)	0.24
Pulmonary oedema	0	0	1	0	0	1	(1)	0	(0)	1.0 (1.0, 1.1)	1
Renal failure	0	0	0	0	0	0	(0)	0	(0)	-	-
Hypoglycaemia	0	0	0	0	0	0	(0)	0	(0)	-	-

*Fisher's Exact Test (2-sided).

**Adjusted for Circulatory Collapse, aOR (95% CI) of Circulatory Collapse = crude OR = 186 (30.4, 1143).

### Treatment

Nearly 90% (345) of suspected severe malaria cases received parenteral therapy on admission which included either an anti-malarial drug alone (238 or 61%), or a combination of anti-malarial and an antibiotic (107 or 27%) (Table 7). Intramuscular artemether was the most frequently administered anti-malarial, given to 324 or 82% of suspected severe malaria patients on admission. Aminopenicillins, ciprofloxacin and cephalosporins were the most frequently given antibiotics.

**Table 7:** Anti malarial and antibiotic treatment on admission versus outcome in probable severe malaria cases (n=393)

** Anti-malarial and antibiotic use**	**Total**		**Died**	
	**n**	**(%)**	**n**	**(%)**
**Only anti malarials**	238	(61)	25	(11)
**Only antibiotics**	18	(5)	2	(11)
**Antibiotics and anti malarials**	107	(27)	22	(21)
**No anti-malarials or antibiotics**	30	(8)	6	(20)
**Number and type of anti malarials**				
At least one anti-malarial	345	(88)	47	(14)
Two or more anti-malarials	15	(4)	2	(13)
Artemether	324	(82)	44	(14)
Artesunate + Mefloquine	13	(3)	0	(0)
Chloroquine	10	(3)	1	(10)
Quinine	9	(2)	3	(33)
Mefloquine	4	(1)	1	(25)
DHA+Piperaquine	2	(1)	0	(0)
DHA	2	(1)	1	(50)
**Number and type of antibiotics**				
At least one antibiotic	125	(32)	24	(19)
Two or more antibiotics	15	(4)	4	(27)
Aminopenicillins	61	(16)	10	(16)
Cephalosporins	37	(9)	14	(38)
Ciprofloxacin	27	(7)	0	(0)
Gentamycin	10	(3)	4	(40)
Co-trimoxazole	4	(1)	0	(0)
Metronidazole	4	(1)	0	(0)
Tetracycline	1	(0)	0	(0)

### Treatment delay, cost and residence according to ARC Zone

Mean time to hospital presentation in severe malaria patients was five days (range one to 30 days) after onset of symptoms. No clear relationship was observed between treatment delay and outcome. Overall CFR of patients originating from ARC Zone 1 districts was lower than those from Zone 2 districts (11% versus 15%) (Table 8). For the majority (45%) of the study population, treatment was subsidized by local NGOs with a fixed US$20 payment, while 38% (150/393) paid for treatment out-of-pocket at a cost of between US$2.5 and 75 (median US$12.5). For 67 patients or 17%, no cost data were available, meaning these patients did not pay or no payment records could be found.

**Table 8:** Case fatality rates and treatment delay versus origin stratified according to artemisinin-resistance containment (ARC) zone for probable severe malaria cases (n=393)

**Province**		**District**						**Onset to presentation (days)**	
**Total**			**Died**		**Survived**		**Total**		
	**n**		**n**	**(%)**	**n**	**(%)**	**n**	**Mean**	**SEM**
Battambang	122	Samlot	11	(10)	101	(90)	112	5.1	0.3
		Phnom Preuk	0	(0)	5	(100)	5	6.0	0.6
		Kam Reang	1	(25)	3	(75)	4	5.5	3.2
		Sampov Loun	0	(0)	1	(100)	1	3.0	-
Pailin	16	Pailin/Sala	3	(19)	13	(81)	16	6.7	1.8
		Kroa							
Pursat	2	Veal Veaeng	1	(50)	1	(50)	2	5.0	2.0
**Total ARC Zone 1**	**140**		**16**	**(11)**	**124**	**(89)**		**5.3**	**0.3**
**Province**		**District**						**Onset to presentation (days)**	
**Total**			**Died**		**Survived**		**Total**		
	**n**		**n**	**(%)**	**n**	**(%)**	**n**	**Mean**	**SEM**
Battambang	241	Ratanak	10	(15)	55	(85)	65	6.0	0.7
		Mondol							
		Banan	8	(18)	37	(82)	45	6.2	0.6
		Battambang	4	(11)	32	(89)	36	5.4	0.6
		Thmar Koul	3	(13)	21	(88)	24	5.2	0.4
		Sangker	5	(24)	16	(76)	21	4.6	0.4
		Moung Russey	3	(17)	15	(83)	18	7.8	1.6
		Ek Phnom	1	(6)	17	(94)	18	5.1	0.6
		Bavel	3	(43)	4	(57)	7	6.7	1.0
		Koh Kralor	1	(14)	6	(86)	7	5.3	1.2
Other	12*		1	(8)	11	(92)		6.7	1.8
**Total ARC Zone 2**	**253**		**39**	**(15)**	**214**	**(85)**		**5.8**	**0.3**

*Banteay Meanchey(4), Pursat(4), Siem Reap(1), Sihanoukville(1), Svay Rieng(1), Takeo(1).

## Discussion

This is one of the few studies to describe the presentation and epidemiology of severe malaria in Western Cambodia, a low transmission setting with multidrug-resistant *P*. *falciparum*, and an increasing predominance of *P*. *vivax* malaria. The study provides baseline information on the burden of severe malaria in a referral hospital near the epicentre of global anti-malarial drug resistance (WHO Zone 1) during the 2.5 years preceding the Artemisinin Resistance Containment Project and supports other preliminary evaluations reporting success in containment and reduction of mortality rates [4]. These intense efforts led by WHO and others provided bed net distribution, and trained local village malaria workers to provide earlier case detection and treatment free of charge, resulting in declines in falciparum malaria cases [5,6]. Following the downward trend in other Containment Project provinces, severe malaria incidence and CFR in Battambang Province reported to the National Malaria Control Program continued to decline significantly after 2009 with only two deaths out of 116 severe cases in 2010 and one death out of 101 severe cases in 2011 [7]. Other likely contributors to the reduction in severe disease in the interim have included malaria case management training, a ban on artemisinin monotherapy, a government crack-down on counterfeit drugs and illegal pharmacies, improved supply of good quality anti-malarials, an increased programme focus on migrant and mobile populations (including the military), and basic improvements in infrastructure such as road access in Western Cambodia. The relationship between level of artemisinin tolerance and pathogenicity of malaria parasites is unclear. High levels of differentiation in artemisinin-resistant *P*. *falciparum* subpopulations have recently been demonstrated in Cambodia [8] and future research may or may not link these to different levels in pathogenicity.

On review of the hospital records, it was often apparent that malaria was a co-existing infection of another serious condition, such as meningitis, sepsis and gastrointestinal haemorrhage which is not unexpected, given the recent evidence of *falciparum* malaria infection as a risk factor for bacteraemia in African children [9]. Bacterial cultures that could have ruled out bacteraemia, were not available at BRH for this population.

Despite substantial limitations, diagnostic and treatment guidelines were reasonably well adhered to where possible at this facility, likely contributing to an overall CFR of 14.2% which was intermediate compared to other reports from the region. CFR in other retrospective studies in the region have varied from 1.8% in Thailand [10], 6.3% in the Philippines [11], to 35% in Malaysia [12]. This compares to recent mortality rates in African studies, largely in children, ranging from 4.5% in Cameroon [13] to 11.5% in Madagascar [14] to up to 22% in children with bacteraemia in Mozambique [6,15]. Despite the emergence of artemisinin resistance in Western Cambodia during the study period, cases of severe malaria and deaths declined in BRH and nationally. Overall CFR was lower in severe malaria patients residing in districts with confirmed artemisinin resistance (Zone 1), likely resulting from increased active case detection and early treatment. Overall malaria incidence in Cambodia increased from 4.1 per 1,000 in 2008 to 6.22 per 1,000 in 2009 attributed to increased migrational movements, while CFR of severe malaria cases continued to decline [2].

Although life-threatening malaria complications can affect patients of all ages, disease presentation and mortality have been found to be closely related to transmission intensity and age of the patient [16]. In a low transmission setting such as this, where immunity may never be acquired, older patients may have higher mortality rates owing to delayed presentation. The most frequent complications associated with mortality, particularly in older patients, were prostration, respiratory distress and coma, with 35% of comatose patients dying on or soon after admission. Coma and metabolic acidosis have been found to be the most significant risk factors for death in both children and adults in prior studies in Asia [17,18]. Others have demonstrated that renal function monitoring in these patients may be crucial for survival [16,19]. The lack of metabolic and chemistry testing inhibited the ability to identify metabolic acidosis and renal dysfunction early, making prognosis and management more challenging. Of the limited diagnostic testing available, mortality did appear to correspond with laboratory abnormalities for both leukocyte and platelet counts, lending some limited prognostic value to these tests. Elevated or depressed leukocyte counts often indicate concomitant bacterial infections, and patients may benefit from empiric antibiotic treatment [20]. Thrombocytopaenia, one of the most common laboratory abnormalities in severe malaria [21] also had some prognostic value for severe disease though it did not predict bleeding complications.

In addition to diagnostic and prognostic limitations, challenges in management included lack of mechanical ventilation units, renal haemodialysis, centrally administered pressors, exchange transfusion capabilities or real time haemodynamic and metabolic data to guide resuscitation. Limited peritoneal dialysis was available at times, but rarely employed. As a result, renal failure and pulmonary oedema, while rare at this facility (reported in only 7% and 2% patients),were associated with the highest CFR in this group.

Recent descriptions of treatment-seeking behaviour, cultural practices and socio-economic barriers have revealed causes for delays in appropriate malaria treatment in Cambodia, representing an important potential area for intervention to reduce mortality [22]. Several factors appear to have contributed to mortality from severe malaria at the BRH including delayed presentation, delayed admission referral, cultural beliefs, and difficulty accessing health care facilities in this austere setting. The average time between onset of symptoms and admission to BRH was 5.5 days, greater in some of the outlying districts. Although Cambodia has an extensive peripheral health centre network, lack of public sector resources may prompt patients to seek initial treatment directly at the highest level of care possible, bypassing outlying networks. The majority of severe cases (70%) presented directly to the BRH, and were admitted directly for treatment to the intensive care unit, with only the minority (approximately 30%) being referred from outlying centres. Referred patients (especially from the public sector) had worse outcomes, suggesting increased treatment delay and lack of pre-referral treatment such as parenteral artemisinins, suppositories and/or transfusion capability.

Roughly 90% of all patients discharged with a severe malaria diagnosis at BRH received intramuscular artemether on the day of admission, which at the time of publication remains the current national standard of care for severe malaria in Cambodia. A five to seven-day course is used, followed by treatment with mefloquine, but is sometimes unavailable, prompting patients to obtain drugs from private-sector pharmacies risking counterfeit or substandard treatment. Parenteral artesunate was not available in Cambodia during the study period. Parenteral artesunate currently remains the most effective treatment remaining for severe malaria [23], and was found to be more effective and less expensive than quinine in children in sub-Saharan Africa [24] and adults in Southeast Asia [25]. Though limited head to head comparisons between intravenous artesunate suggest some degree of inferiority for intramuscular artemether [26], both were found to be effective, and the relative simplicity of rapid intramuscular administration in critical illness may support its continued use in Cambodia. Availability and frequent of use of this life-saving drug may explain the relatively low overall mortality rates seen, particularly considering the limited array of diagnostic and treatment facilities available. Quinine continues to be the first-line agent for pregnant women in Cambodia.

There has been recent recognition of the substantial proportion of severe malaria cases attributable to *P*. *vivax* in tropical regions [27]. Observed rates of severe *P*. *vivax* in Southeast Asia and the Pacific have been high in several reports, accounting for up to 25% of severe malaria cases. While some have shown reduced risk of severe disease attributable to *P*. *vivax* and/or mixed infection in low transmission settings [28], others have shown risks of severe disease to be increased, particularly in young children and in areas with high grade chloroquine resistance, though the latter has not been reported in Cambodia [29]. Though *P*. *vivax* is typically thought of as a relatively benign disease, 14.5% of patients meeting the case definition of severe malaria at BRH during the study period had slide proven *P*. *vivax*, and there were four documented deaths. There were no deaths reported among 17 patients with documented mixed infection. HIV-1 infection has been reported to be an important risk factor for severe malaria and death [30], but there was only one confirmed case of HIV co-infection in this study population.

The study’s retrospective design placed limits on accurately interpreting the underlying disease burden. Accurately defining the terms ‘impaired consciousness’ and ‘prostration/coma’ retrospectively were challenging. WHO defines impaired consciousness as a ‘rousable mental condition’, and prostration as the inability to sit upright without support or drink. However, varying terms were used by clinicians at BRH, including descriptions such as “somnolence”, “obnubilation”, “agitation”, “delirium”, “irritation”, “pre-coma”, and in some cases a Glasgow Coma Score (GCS) was given. In the retrospective analysis, the study team adopted the term ‘coma’ for those found to have either a clearly documented coma state, or understood to have an unarousable coma based on GCS scores. Limitations on diagnostic capabilities at this facility leaves open the possibility that cases and/or complications may have been missed. The study does not reflect cases that never made it to medical attention, died out of hospital, or were treated at local facilities, in the private sector or at home. As such, the in-patient data are only representative of the referral centre and not of the overall severe malaria burden for this region of Cambodia. Likewise, the non-availability of important diagnostic measurements may have resulted in under-reporting of complications.

A large proportion of patients were slide-negative on admission, though nearly 50% of these patients still responded to anti-malarials alone. In such cases, preliminary microscopic diagnosis in hospital may have been obscured or simply not performed due to pre-treatment out of hospital prior to transfer. Many cases were diagnosed by rapid diagnostic kits or simply treated on clinical grounds. Patients with negative malaria smears in this cohort had higher crude mortality rates at 16% versus 11% in the patients with high parasitaemia, possibly due to delays in diagnosis and appropriate treatment, or earlier parenteral therapy in the higher parasitaemia patients. In this setting, a high index of suspicion for severe malaria remains warranted and may prove lifesaving. However, an empiric treatment approach to severe malaria should not preclude investigation of co-morbidities, which were common.

## Conclusions

BRH continued to have a significant number of severe malaria cases between 2006–2009, suggesting that interventions to improve access to early diagnosis and treatment of malaria remain priorities for this region. Diagnostic ambiguities were common in this setting, with multiple confounding co-morbidities and limited patient management resources. The key to preventing mortality remains early detection, treatment and referral where necessary, and maintaining a high index of suspicion for severe malaria cases is essential. This includes not only adherence to guidelines, but consideration of the individual patients’ risks based on local endemicity, clinical history, pre-hospital care, and severity of presenting illness syndromes. Co-administration of broad-spectrum antibiotics in this resource-poor setting with frequent co-infection and septicaemia may improve outcome. A subset of technologically appropriate core guidelines for diagnosis and management of severe malaria may be useful in this setting, and monitoring of the patient’s renal and acid–base balance through simple chemistry panels may be a useful and cost-effective addition to management at this and similar facilities. The drop in both numbers of severe cases and mortality during the period immediately following this study suggests some improvement in malaria control, likely to due to large-scale containment efforts in the region.

## Competing interests

The authors declared that they have no competing interests.

## Authors’ contributions

DS, CL, AT, DB, JL, YS, NS, KL, ST, MF, DW and CMC conceived, designed and supported the study. NB, SN, SC, CL, DS and YS collected the data. AT, CL, DS, SK, TW, DC and SJ analysed the data. DS, AT, DB and CL wrote the paper. YS passed away during the writing of this manuscript. All authors read and approved the final manuscript.
